# Antibodies to Citrullinated Proteins (ACPA) Associate with Markers of Osteoclast Activation and Bone Destruction in the Bone Marrow of Patients with Rheumatoid Arthritis

**DOI:** 10.3390/jcm10081778

**Published:** 2021-04-19

**Authors:** Weronika Kurowska, Iwona Slowinska, Zbigniew Krogulec, Piotr Syrowka, Wlodzimierz Maslinski

**Affiliations:** 1 Department of Pathophysiology and Immunology, National Institute of Geriatrics, Rheumatology and Rehabilitation, Spartanska 1, 02-637 Warsaw, Poland; zaklad.patofizjologii@spartanska.pl; 2 Department of Rheumoorthopaedic Surgery, National Institute of Geriatrics, Rheumatology and Rehabilitation, Spartanska 1, 02-637 Warsaw, Poland; iwona.slowinska@spartanska.pl (I.S.); zbigkrogi@wp.pl (Z.K.); piotr.syrowka@spartanska.pl (P.S.)

**Keywords:** rheumatoid arthritis, ACPAs, anti-CCP antibodies, bone destruction, bone marrow, osteoimmunology

## Abstract

Normalizing bone metabolism is a challenge in rheumatoid arthritis (RA). Studies in mice suggest that anti-citrullinated protein antibodies (ACPAs) can trigger osteoclast activation and bone resorption in the bone marrow. However, data on the presence and role of ACPAs in human bone marrow are scarce. We investigated whether ACPAs can contribute to osteoclast activation and bone erosion in RA bone marrow. Anti-cyclic citrullinated peptide antibodies (anti-CCP Abs), osteoclast activation indicators–the tartrate-resistant acid phosphatase 5b (TRAP5b) and cathepsin K, and bone degradation marker–C-terminal telopeptide of type I collagen (CTX-I) were measured in the bone marrow and peripheral blood of RA patients using ELISAs. We found that ACPAs present in RA bone marrow was associated with increased amounts of TRAP5b, cathepsin K and CTX-I in this location. Levels of IL-8, the key mediator of anti-citrullinated protein antibody (ACPA)-induced bone resorption, were also elevated in bone marrow containing anti-CCP Abs and positively correlated with TRAP5b and cathepsin K concentrations. Higher levels of TRAP5b, cathepsin K, CTX-I and IL-8 in bone marrow compared to peripheral blood indicate local generation of these molecules. Our results complement data from animal studies and highlight the relevance of ACPAs and bone marrow in bone resorption in RA.

## 1. Introduction

Rheumatoid arthritis (RA) is the most common autoimmune joint disease, affecting 0.5–1% of adults in developed countries [1]. The disease is characterised by chronic inflammation of the synovial membrane (synovitis) and the progressive destruction of articular cartilage and bone. Current therapeutic strategies for RA interfere with inflammation-related processes, and some of them inhibit the progression of bone destruction but unfortunately have little or no effect on bone repair [2,3]. Consequently, the disease progresses, leading to joint dysfunction and ultimately, disability. Therefore, the important therapeutic challenge is to normalize bone metabolism in RA. The process of bone destruction in RA results from the hyperactivation of osteoclasts, leading to increased bone resorption, traditionally thought to be due to the paracrine action of cytokines released from the inflamed synovium [4]. However, a growing amount of data indicate that bone marrow also plays an essential role in osteoclast activation and bone destruction in RA. In a mouse model of arthritis, bone marrow pathologies include increased osteoclast differentiation and accumulation [5,6]. In patients with RA, the overexpression of osteoclastogenesis-promoting factors and active de novo forming osteoclasts have been revealed in bone marrow [7,8,9]. In addition, bone marrow edema, reflecting inflammatory infiltrates in this tissue, is associated with the progression of bone erosion and is a predictor of bone destruction in RA [10,11,12].

Recent animal studies suggest that bone marrow may be an independent or even primary site of bone destruction in RA and show that anti-citrullinated protein antibodies (ACPAs)–the most specific serological marker of RA, may be involved in this process [13,14,15]. It has been shown that ACPAs isolated from RA patients can stimulate murine and human osteoclast precursor cells and mature osteoclasts, leading to their differentiation and enhancement of effector functions: bone resorption and production of inflammatory mediators in vitro [13,14,15]. Intriguingly, ACPAs that were transferred to mice, bound to bone marrow-resident osteoclasts and osteoclast precursor cells and led to joint pain and systemic bone loss prior to the development of synovitis [13,14]. The existence of this novel mechanism of bone resorption could explain why bone destruction in RA progresses despite the absence or suppressed inflammation (in healthy anti-citrullinated protein antibody (ACPA)-positive individuals or patients achieving sustained clinical remission) and why the first bone lesions appear in the cortical bone, at the border of the joint and bone marrow [16,17]. Further analyses have identified extracellular interleukin-8 (IL-8) as the key mediator of ACPA-triggered osteoclastogenesis and bone loss in mice. Blocking this cytokine activity completely reversed the pathogenic effects of ACPAs in vitro and in vivo [13,14].

In humans, ACPAs are recognized as a strong risk factor for bone destruction [18,19,20]. However, data on their presence and role in the human bone marrow are scarce. In this study, we examined whether ACPAs and IL-8 can be relevant for osteoclast activation and bone resorption in the bone marrow of RA patients.

## 2. Materials and Methods

### 2.1. Patients and Material

The study involved 42 patients with RA (according to the ACR 1987-revised classification criteria [21]). Bone marrow samples taken from the femur during a hip replacement surgery performed as part of normal clinical care. Paired peripheral blood samples were collected 1–1.5 h before the hip joint prosthesis implantation. All patients gave written informed consent to participate in the study. The study was approved by the Ethics Committee at the National Institute of Geriatrics, Rheumatology and Rehabilitation in Warsaw, Poland (approval code KBT-1/4/2019).

### 2.2. Measurement of ACPA, IL-8, TRAP5b, Cathepsin K and CTX-I

Samples of bone marrow and peripheral blood plasma were isolated by centrifugation of the whole bone marrow/ peripheral blood, as previously described [7]. For the measurement of ACPAs, a diagnostic EDIA test detecting IgG antibodies against cyclic citrullinated peptides (anti-CCP Abs) from EuroDiagnostica (Malmö, Sweden) was used according to the manufacturer’s instructions. Commercial enzyme immunoassays were used to quantify the concentrations of IL-8 (Quidel, Athens, OH, USA), the tartrate-resistant acid phosphatase 5b isoform–TRAP5b (BioVendor, Brno, Czech Republic), cathepsin K and C-terminal telopeptide of type I collagen–CTX-I (last two from Cloud-Clone, Katy, TX, USA).

### 2.3. Statistical Analyses

Data were analyzed using the Statistica Software vol. 6.0 (StatSoft, Cracow, Poland). The normality of the data was assessed using the Shapiro–Wilk test, and then the differences between groups were evaluated using the appropriate test (two-tailed Student’s *t*-test or two-tailed Mann–Whitney U test). Spearman’s rank correlation coefficient was used to measure the relationship between the variables. *p* values less than 0.05 were considered significant.

## 3. Results

### 3.1. Presence of ACPA in Bone Marrow of RA Patients

We investigated bone marrow plasma samples from patients diagnosed with ACPA-positive RA or ACPA-negative RA based on the presence (or absence, respectively) of anti-CCP Abs in their peripheral blood. Our analyzes showed that all patients who had anti-CCP Abs detected in the peripheral blood during the diagnostic process also had anti-CCP Abs in their bone marrow. Patients without anti-CCP Abs in peripheral blood also did not contain anti-CCP Abs in their bone marrow. In the studied population, the mean concentration of anti-CCP Abs in the bone marrow plasma was 5916.0 ± 8283.0 U/mL (range 0.0–29804.0). Thus, we demonstrated that in anti-CCP Abs-positive RA patients, ACPAs are present in the bone marrow compartment. The clinical characteristics and demographic data of the patients are presented in Table 1.

### 3.2. Elevated Levels of, TRAP5b, Cathepsin K and CTX-I in ACPA-Positive Bone Marrow

**Figure 1:**
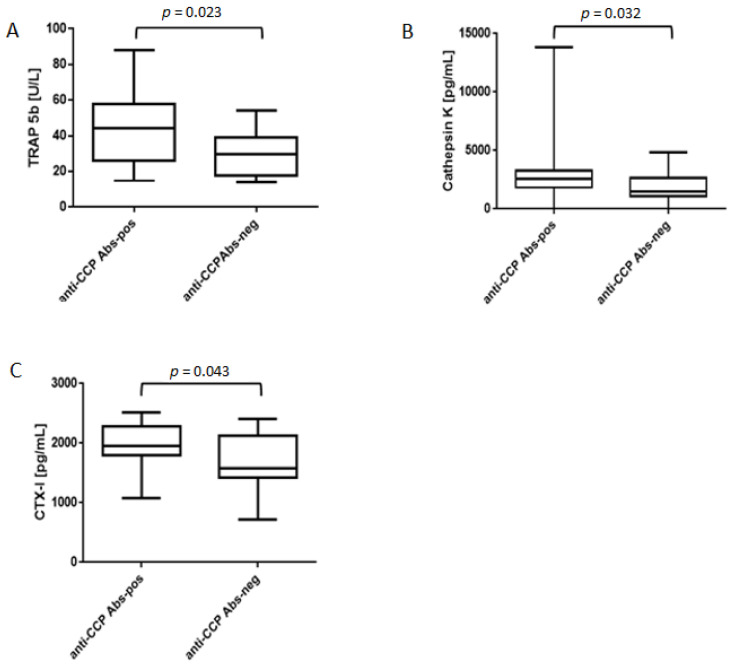
Concentrations of TRAP5b, cathepsin K and CTX-I in the bone marrow of RA patients. (**A**–**C**) Anti-cyclic citrullinated peptides antibodies-positive (anti-CCP Abs-pos)/anti-cyclic citrullinated peptides antibodies-negative (anti-CCP Abs-neg) bone marrow samples: *n* = 20/18 (**A**), *n* = 19/17 (**B**) and *n* = 22/20 (**C**). Comparisons were performed using two-tailed Student’s *t*-test (A and C) or two-tailed Mann–Whitney U test (**B**). TRAP5b, tartrate-resistant acid phosphatase 5b; CTX-I, C-terminal telopeptide of type I collagen.

Next, we evaluated the level of osteoclast activity and the degree of bone resorption in bone marrow from RA patients. For this purpose, we measured the concentrations of osteoclast activation indicators (TRAP5b and cathepsin K) and bone resorption indicator (CTX-I) in bone marrow plasma. Our analyzes showed the presence of all three tested osteoclast activation/bone resorption indices in both types of bone marrow samples: anti-CCP-positive and anti-CCP-negative. However, the concentrations of TRAP5b, cathepsin K and CTX-I were significantly higher in the bone marrow that contained anti-CCP Abs (Figure 1). The mean concentrations of TRAP5b in anti-CCP Abs-positive samples and anti-CCP Abs-negative samples were 43.6 ± 20.2 U/L and 30.4 ± 12.5 U/L, respectively. Regarding cathepsin K, the median concentrations of this enzyme were 2574.0 pg/mL (range 0.0–13821.0) in anti-CCP Abs-positive samples and 1478.0 pg/mL (range 0.0–4838.0) in anti-CCP Abs-negative samples. The mean quantities of CTX-I, the degradation product of bone collagen, in the bone marrow were as follows: 1979.0 ± 370.5 pg/mL in anti-CCP Abs-positive samples and 1719.6 ± 435.4 pg/mL in anti-CCP Abs-negative samples. Our results indicate increased osteoclast activity and bone resorption in the RA bone marrow, which contains anti-CCP Abs.

### 3.3. Elevated Levels of IL-8 in ACPA-Positive Bone Marrow

**Figure 2:**
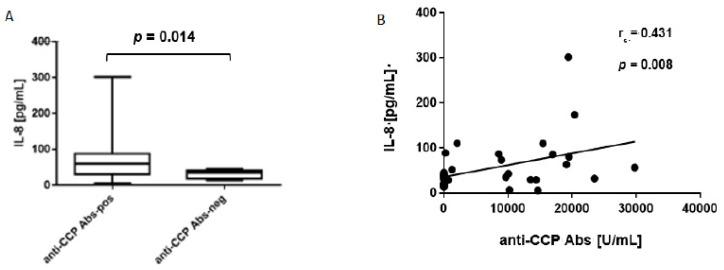
Concentrations of IL-8 in the bone marrow of RA patients. (**A**) Concentrations of IL-8 in anti-cyclic citrullinated peptides antibodies-positive (anti-CCP Abs-pos), *n* = 20, and anti-cyclic citrullinated peptides antibodies-negative (anti-CCP Abs-neg), *n* = 17, bone marrow samples. Comparison was performed using two-tailed Mann–Whitney U test. (**B**) Correlation between IL-8 and anti-CCP Abs concentrations, *n* = 37. The relationship between variables was measured using the Spearman’s rank correlation coefficient.

Further analyses showed higher median concentrations of IL-8 in anti-CCP Abs-positive than in anti-CCP Abs-negative bone marrow samples (60.1 pg/mL, range 6.0–301.4, vs 34.4 pg/mL, range 13.4–45.4, respectively) (Figure 2A). Moreover, the concentration of IL-8 correlated with the level of anti-CCP Abs in the bone marrow (Figure 2B). Our findings indicate that amount of IL-8 in the bone marrow of RA patients is related to the presence of ACPAs.

### 3.4. Levels of IL-8 Associate Positively with TRAP5b, Cathepsin K and CTX-I Concentrations in ACPA-Positive Bone Marrow

**Figure 3:**
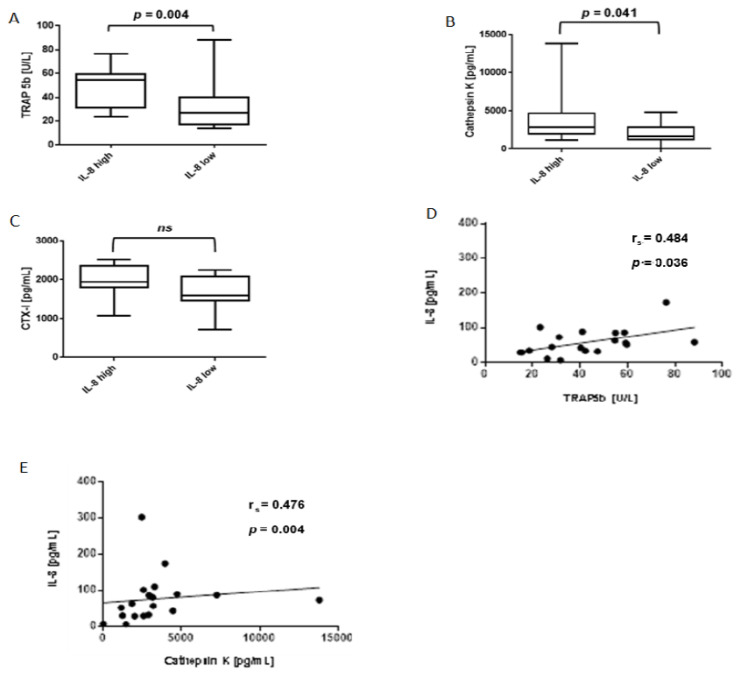
Concentrations of TRAP5b, cathepsin K and CTX-I in bone marrow containing high or low levels of IL-8. (**A**–**C**) Levels of TRAP5b, cathepsin K and CTX-I in IL-8-low and IL-8-high bone marrow samples (description in the text), *n* = 23/11; Comparisons were performed using two-tailed Mann– Whitney U test; ns, not significant; (**D–E**) Correlation between IL-8 and TRAP5b (**D**), and cathepsin K (**E**) concentrations in anti-CCP Abs-positive bone marrow, *n* = 19; the relationships between variables were measured using the Spearman’s rank correlation coefficient. TRAP5b, tartrate-resistant acid phosphatase 5b; CTX-I, C-terminal telopeptide of type I collagen.

To investigate whether the presence of IL-8 can be related to osteoclast activation in RA bone marrow, we examined the association of IL-8 with the levels of osteoclast activity markers–TRAP5b and cathepsin K in this location. We found increased median concentrations of both TRAP5b and cathepsin K in IL-8-rich bone marrow samples (i.e., containing more than 51.5 pg/mL IL-8, which is the threshold value corresponding to the average amount of IL-8 in the bone marrow calculated for the entire study population; IL-8-high samples) (Figure 3A,B, respectively). The TRAP5b concentrations were 54.79 U/L (range 27.1–76.3) in IL-8-high bone marrow samples and 28.2 U/L (range 14.0–88.2) in the bone marrow samples containing lower levels of IL-8 (IL-8-low samples) (Figure 3A). Cathepsin K concentrations were 2918.0 pg/mL (range 1149.3–13821.0) and 1660.0 pg/mL (range 0.0–4837.7) in IL-8-high and IL-8-low bone marrow samples, respectively (Figure 3B). We also observed a higher median level of CTX-I in IL-8-high bone marrow samples (1945.0 pg/mL, range 1174.0–2510.0) than that found in IL-8-low bone marrow samples (1600.0 pg/mL, range 712.0–2256.0), but this result was not statistically significant (Figure 3C). In addition, we found a positive correlation of the levels of TRAP5b and cathepsin K with IL-8 concentration in bone marrow containing anti-CCP Abs (Figure 3D,E, respectively). We did not observe a correlation between IL-8 and TRAP5b or cathepsin K levels in anti-CCP Abs-negative bone marrow samples. The results of these analyses indicate a positive association between IL-8 concentration and osteoclast activity in RA bone marrow, which contains anti-CCP Abs.

### 3.5. Higher Levels of IL-8, TRAP5b, Cathepsin K and CTX-I in Bone Marrow Than in Peripheral Blood od ACPA-Positive RA Patients

**Figure 4:**
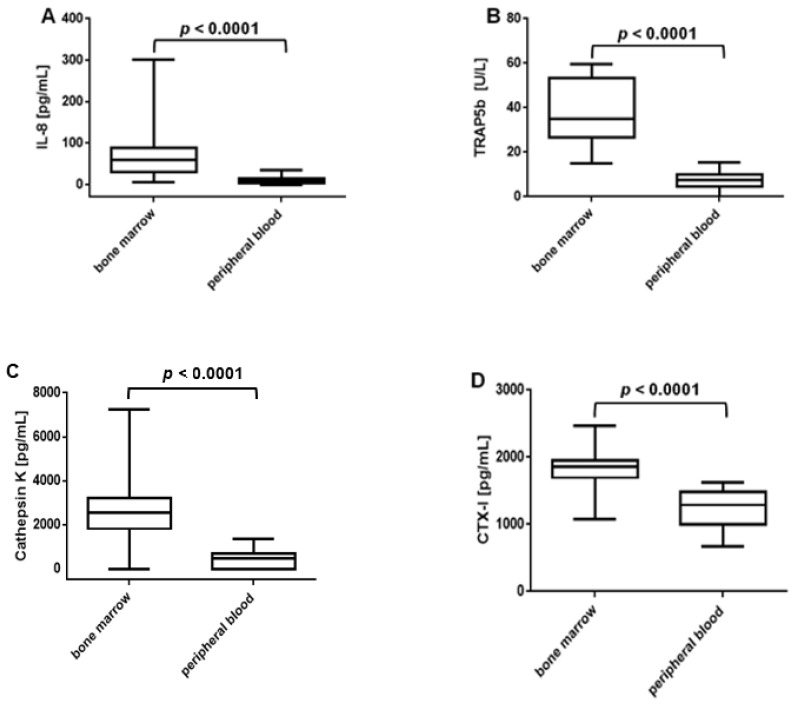
Concentrations of IL-8, TRAP5b, cathepsin K and CTX-I in paired bone marrow and peripheral blood samples of ACPA-positive RA patients. (**A**–**D**) Bone marrow/peripheral blood samples: *n* = 16 (**A**), *n* = 16 (**B**), *n* = 14 (**C**), *n* = 15 (**D**). Comparisons were performed using two-tailed Mann–Whitney U test (**A**,**C**) or two-tailed Student’s *t*-test (**B**,**D**). TRAP5b, tartrate-resistant acid phosphatase 5b; CTX-I, C-terminal telopeptide of type I collagen; ACPA, anti-citrullinated protein antibodies.

We then compared the amounts of IL-8, TRAP5b, cathepsin K and CTX-I in the bone marrow and peripheral blood of anti-CCP Abs-positive RA patients. We found significantly higher concentrations of each of these factors in the bone marrow than in the peripheral blood (Figure 4). The median concentrations of IL-8 were as follows: 60.1 pg/mL (range 6.0–301.4) in the bone marrow and 10.3 pg/mL (range 0.0–35.7) in peripheral blood (Figure 4A). The mean concentrations of TRAP5b in the bone marrow and in the peripheral blood were 38.2 ± 14.3 U/L and 7.3 ± 3.3 U/L, respectively (Figure 4B). Cathepsin K median concentration was 2566.0 pg/mL (range 0.0–7256.0) in bone marrow and 176.0 pg/mL (range 0.0–1346.0) in peripheral blood (Figure 4C). As for the mean concentration of CTX-I, it was found to be 1812.0 ± 183.7 pg/mL in the bone marrow and 1236.0 ± 80.0 pg/mL in peripheral blood (Figure 4D). Thus, our observations indicate that the bone marrow of anti-CCP Abs-positive RA patients can be a site of IL-8, TRAP5b, cathepsin K and CTX-I generation.

## 4. Discussion

Pathological bone erosion begins early in RA—in the first months of the clinical disease or even before the onset of clinical symptoms [16,22,23,24]. This suggests the existence of an additional, at least partially independent from synovial inflammation, mechanism for osteoclast stimulation and bone destruction in RA. In addition to their diagnostic significance, antibodies to citrullinated proteins may also be involved in promoting several pathological processes in RA [25]. However, their role in bone destruction is not fully understood. Interestingly, the appearance of ACPAs in serum is also an early hallmark of RA, as these antibodies can emerge up to two decades before diagnosis [26,27]. Moreover, the observation that systemic bone loss and cortical bone loss occur in healthy subjects with ACPA positivity suggests that ACPAs may directly trigger bone destruction in RA [16].

Proposed by two independent research groups, a novel mechanism of ACPA-induced bone erosion independent of synovitis may explain why and how the development of ACPAs is related to joint pathology. As demonstrated in animal models of arthritis, this mechanism can operate from the bone marrow side [13,14,15]. The results of imaging studies carried out in patients with RA also support the concept of the role of bone marrow in the process of bone destruction. Data from Doppler ultrasound and MRI showed an association between the progression of bone erosions and the occurrence of bone marrow edema in RA that was independent of synovial inflammation [12,28]. Furthermore, the simultaneous occurrence of bone marrow edema and ACPAs in the developmental phase of RA, i.e., in patients with undifferentiated arthritis, is strongly associated with progression to full-blown RA [29].

In this study, we demonstrated the presence of ACPAs in the bone marrow of RA patients. The source of ACPAs in established RA may be the synovium, but these antibodies also may originate from the lung or/and periodontal tissue [30,31]. In RA, ACPAs can also be produced by long-lived plasma cells residing in the bone marrow [32].

Our next finding was that ACPAs are associated with increased concentrations of osteoclast activity markers and bone resorption indicators in the bone marrow of RA patients. The levels of the active TRAP5b isoform, reflecting the number and functionality of osteoclasts [33,34], and cathepsin K, which is a cysteine protease that degrades bone matrix proteins and is expressed by mature resorbing osteoclasts [35,36], were significantly higher in the bone marrow samples containing ACPAs than in those without ACPAs. The ACPA-positive bone marrow samples also contained increased concentrations of the bone collagen degradation product CTX-I, which is produced as a result of the enzymatic activity of cathepsin K [37]. Comparisons of the amounts of TRAP5b, cathepsin K and CTX-I in the bone marrow and peripheral blood revealed much higher concentrations of investigated molecules in the bone marrow. This observation points to the role of the bone marrow in the induction/enhancement of bone resorption in RA. Collectively, our results indicate enhanced osteoclast activation in the bone marrow when ACPAs are present at this location.

The crucial mediator of ACPA-driven osteoclastogenesis is IL-8 [13,14]. The concentration of this cytokine increases during M-CSF and RANKL-induced differentiation of osteoclasts and rises additionally in the presence of ACPAs. Importantly, neutralization of IL-8 by specific Ab (but not neutralization of anti-TNF-α) in the presence of M-CSF and RANKL was sufficient to block osteoclasts differentiation in vitro [13]. Furthermore, the application of IL-8 antagonist reparixin was effective in reversing ACPA-induced bone loss in mice in vivo [14]. Thus, IL-8 seems to be necessary and sufficient for pathological osteoclasts development and activity induced by ACPAs in animal models of arthritis. However, research using murine models has inherent limitations as murine models do not entirely represent human disease [38]. Hence, also treatment effects in murine model may differ from those observed in humans. It was shown that the application of intermittent parathyroid hormone, an anabolic agent for bone, in combination with TNF inhibitor led to the significant repair of joint erosions in the TNF-transgenic murine models of RA [39], but not in established RA [40]. Therefore, the results of studies in mice need to be further confirmed in humans. Our analyses of bone marrow samples from patients with RA revealed increased IL-8 levels in ACPA-positive bone marrow. This suggests that ACPAs can be involved in the production of IL-8 in bone marrow in RA. Moreover, we found that osteoclast activity reflected by TRAP5b and cathepsin K levels correlates positively with IL-8 concentration in ACPA-positive bone marrow. These findings are consistent with the trend towards more intensive bone collagen degradation in RA bone marrow containing high levels of IL-8, as assessed based on CTX-I level. Taken together, our results showed the relationship between ACPAs, IL-8 and osteoclast activity in the bone marrow of RA patients. This indicates the relevance of ACPAs and IL-8 in osteoclast activation and bone resorption in the bone marrow tissue in RA, which is consistent with in vitro studies and in vivo observations from animal studies. Interestingly, the contribution of bone marrow in arthritis-related osteoclastogenesis has recently been highlighted, showing that the osteoclasts in the inflamed synovium originate exclusively from circulating bone marrow-derived precursors and not from local macrophages [41].

Our study has several limitations. First, we investigated patients with advanced stages of the disease. Thus, in this study, we were unable to answer the question of whether ACPA-driven bone destruction in the bone marrow may precede and then lead to synovitis in humans, as assumed in the RA model with a central role of bone marrow [13]. This scenario is supported by the appearance of changes in the bone marrow and their coexistence with ACPAs already in the early stages of RA development [29]. If it is so, the early selective intervention to interrupt the functioning of this mechanism, in conjunction with anti-inflammatory therapy, might be of benefit to RA patients [13,42]. The advanced stage of the disease in our patients was also the reason that we could not exclude in this study the possible influence of pro-inflammatory mediators derived from the affected synovium on osteoclast stimulation in the bone marrow. It is worth mentioning here that the bone marrow is also the site of pro-inflammatory cytokines production in the course of RA [5,43,44]. Furthermore, ACPAs are known as stimulators of several inflammatory mediators’ expression in vitro and in vivo [13,14,15]. The other limitation is a relatively small number of subjects studied. Therefore, our results should be further validated in other research centers and larger cohorts of patients.

To summarize, we found that increased levels of osteoclast activation and bone resorption markers TRAP5b, cathepsin K and CTX-I are associated with the presence of ACPAs in the bone marrow of RA patients. Furthermore, the concentration of IL-8, an important factor in the mechanism of ACPA-dependent bone resorption, correlated positively with the level of ACPAs in the bone marrow of patients with RA. We also observed a positive relationship between IL-8 levels and TRAP5b, cathepsin K and CTX-I concentrations in ACPA-containing bone marrow. Our results complement data from animal studies, highlighting the importance of ACPAs and IL-8 in bone destruction in RA patients. From a clinical perspective, our findings support the concept that interference with the production/activity of IL-8 and ACPAs may have benefits in inhibiting/preventing bone erosion, particularly in ACPA-positive RA patients and ACPA-positive individuals at risk of developing RA. In addition, our results advocate that bone marrow should be taken into consideration when planning therapeutic interventions aiming to not only dampen inflammation but also osteoclast activation, especially in ACPA-positive RA patients.

## Figures and Tables

**Table 1:** Patient’s characteristics.

	ACPA-Positive ^1^ (*n* = 22)	ACPA-Negative ^1^ (*n* = 20)
Age (years), median (range)	59 (49–69)	60 (45–70)
Sex, female/male	19/3	17/3
ESR ^2^ (mm/h), mean ± SD ^3^	32.5 ± 16.7	21.0 ± 16.9
CRP ^4^ (mg/L), median (range)	10.5 (1–65)	8 (1–56)
DAS28 ^5^	3.76 ± 0.24	3.62 ± 0.51
Methotrexate/Sulfasalazine/Leflunomide/Azathioprine	21	17
Steroids	17	11
Biologics ^6^	7	5

^1^ based on the presence of anti-cyclic citrullinated peptides antibodies (anti-CCP Abs) in the peripheral blood; ^2^ ERS, erythrocyte sedimentation rate; ^3^ SD, standard deviation; ^4^ CRP, C-reactive protein; ^5^ disease activity score 28; ^6^ discontinued at least one year prior to study. ACPA, anti-citrullinated protein antibodies.

## Data Availability

The data presented in this study are available on request from the corresponding author.
